# Urgent Need to Strengthen the Chain of Survival in the United Arab Emirates; a Letter to the Editor

**Published:** 2017-01-14

**Authors:** Alan Michael Batt, Ahmed Saleh Mohamed Al-Hajeri, Fergal Henry Cummins

**Affiliations:** 1. National Ambulance LLC, Abu Dhabi, UAE.

Further to our recently published findings, we have completed the analysis of our second year of prehospital data collection for the same service (1). We wish to expedite the availability of this data to clinicians, researchers and policy makers in the region.

Our 2015/2016 out-of-hospital cardiac arrest (OHCA) data displayed the following demographic results: 514 OHCA resuscitation attempts were attended by national ambulance (NA) emergency medical services (EMS) in the Northern Emirates region (75% male). Male patients continued on average to be younger than female ones (50 vs. 61 years), and the median age of OHCA cases in the United Arab Emirates remains well below that of cases in Western countries (52 years, interquartile range: 38; 69).

Over half of these cases occurred at a home residence, with the next most common location being a street or highway. A total of 282 (54.5%) incidents were witnessed by a bystander, 43 (8.3%) events by NA crew, and 189 (36.7%) incidents were not witnessed. Bystander cardiopulmonary resuscitation (CPR) was attempted in 135 (28.6%) of non-EMS-witnessed cases (n=471). A bystander or public access defibrillator was applied in only five cases (1%) and no shocks were delivered by bystanders in any case. A total of 34 (6.6%) patients had a return of spontaneous circulation in the pre-hospital setting, over twice the rate demonstrated in the first year of our study. Survival to discharge data has been collected for the first time by our hospital partners, and the publication of these results in the near future will contribute greatly to our understanding of the OHCA issue in the region.

The increase in the number of OHCA responses by NA crew is notable in our findings. This may be attributable to several reasons, including greater public awareness of EMS capabilities, and improved access to EMS via the dedicated 998 emergency number and the NA mobile application. This trend is to be welcomed, as implementation of the chain of survival increases the odds for survival (2). However, similar to our previous findings, a large number of the cases that were witnessed still had a significant time lapse before EMS was activated.

A chain is only as strong as its weakest link. As such, increasing public awareness of the need for early EMS activation, improving bystander CPR rates, and increasing the availability of public access defibrillators still remain significant challenges in implementing the chain of survival in full to address this public health issue in the United Arab Emirates.
